# Fast Prediction Method for Scattering Parameters of Rigid-Flex PCBs Based on ANN

**DOI:** 10.3390/s24072221

**Published:** 2024-03-30

**Authors:** Jingling Mei, Haiyue Yuan, Xinxin Guo, Xiuqin Chu, Lei Ding

**Affiliations:** 1 Shanghai Institute of Technical Physics, Chinese Academy of Sciences, Shanghai 200083, China; meijingling@mail.sitp.ac.cn; 2 School of Electronic, Electrical and Communication Engineering, University of Chinese Academy of Sciences, Beijing 100049, China; 3 Key Laboratory of Infrared System Detection and Imaging Technologies, Shanghai Institute of Technical Physics, Chinese Academy of Sciences, Shanghai 200083, China; 4 Key Laboratory of Ultra High Speed Circuit Design and Electromagnetic Compatibility, Ministry of Education, Xidian University, Xi’an 710071, China; haiyueyuan@stu.xidian.edu.cn (H.Y.); 21021211039@xidian.edu.cn (X.G.); xqchu@mail.xidian.edu.cn (X.C.)

**Keywords:** scattering parameters, InGaAs detection systems, BP neural network, rigid-flex boards, grid ground plane

## Abstract

InGaAs detection systems have been increasingly used in the aerospace field, and due to the high signal-to-noise ratio requirements of short-wave infrared quantitative payloads, there is an urgent need for methods for the rapid and precise evaluation and the optimal design of these systems. The rigid-flex printed circuit board (PCB) is a vital component of InGaAs detectors, as its grid ground plane design parameters impact parasitic capacitance and thus affect weak infrared analog signals. To address the time-intensive and costly nature of design optimization achieved with simulations and experimental measurements, we propose an innovative method based on a neural network to predict the scattering parameters of rigid-flex boards for InGaAs detection links. This is the first study in which the effects of rigid-flex boards on weak infrared detection signals have been considered. We first obtained sufficient samples with software simulation. A backpropagation (BP) neural network prediction model was trained on existing sample sets and then verified on a rigid-flex board used in a crucial aerospace short-wave infrared quantitative mission. The model efficiently and accurately predicted high-speed interconnect scattering parameters under various rigid-flex board grid plane parameter conditions. The prediction error was less than 1% compared with a 3D field solver, indicating an overcoming of the iterative optimization inefficiency and showing improved design quality for InGaAs detection circuits.

## 1. Introduction

In recent decades, short-wave infrared InGaAs focal plane array detectors [1] have been extensively used in aerospace remote sensing. With the continuous improvement in remote sensing payload indicators, the total number of remote sensing data has increased rapidly [2]. It has thus become common to see the data transfer capacity of high-resolution satellites in the order of GHz, a trend that in turn has led to the increased use of large-area array detectors and a corresponding increase in readout frequency. Quantification, which is essential in quantitative payloads, requires an improvement in signal fidelity. However, in practice, there is often a need to balance the design requirements of the optical system with those of the mechanical structure. Generally, the InGaAs detectors cannot be directly coupled to the driver acquisition circuit board. However, rigid-flex boards, with their compact, lightweight, and highly reliable mechanical structure [3], play a vital role in the connection between short-wave infrared photodetectors [4] and information acquisition circuits. Nevertheless, high-speed signal transmission on rigid-flex boards can significantly distort infrared analog signals [5] before they reach the driver acquisition module, presenting potential signal integrity challenges in a low-noise infrared detection system design. To enable accurate quantization under high signal-to-noise ratio requirements, it is imperative to design each component of the detection link precisely. Therefore, modeling and optimizing the transmission characteristics of rigid-flex boards are necessary.

Grid ground planes are quite commonly used in a modern rigid-flex PCB design to meet the requirements of impedance matching and interlayer interconnections [6,7,8,9,10]. Traces over the grid ground plane are associated with signal integrity (SI) degradation, electromagnetic interference (EMI), and ground bounce in general [11]. Hence, it is vital to investigate how the electrical properties of a transmission line change as a function of the geometry of the grid ground planes. Scattering parameters (S-parameters), as a beneficial means for describing the behavior of linear passive electrical properties, contain much information about interconnect transmission characteristics such as the impedance, crosstalk, reflection, and attenuation of the transmitted signals [12]. Moreover, recent studies on rigid-flex PCBs have focused on how various design parameters in grid grounding can be applied to impedance matching [13,14,15], but how this affects the S-parameters has not yet been investigated. To achieve optimal performance in the signal link transmission, the link design parameters must be adjusted repeatedly for S-parameter optimization, which can improve rigid-flex PCBs’ analysis and optimization capabilities in infrared fine detection systems, resulting in better design effects and lower noise levels.

In the past, S-parameters were frequently obtained in the early design phase with time-consuming and labor-intensive experimental measurements [16,17,18,19] which required precise test equipment. It is now widely recognized that the transmission line referenced to a grid ground plane is a periodically varying structure [20,21]. Therefore, with the advancement of modern electronic design automation (EDA), 3D field solvers [22,23] have been widely adopted to simulate experimental measurements. Substituting expensive and time-consuming physical measurements with simulated results obtained with 3D field solvers significantly reduces the cost and design cycle associated with fabrication and measurements. Macromodeling is a fundamental tool for the characterization of high-frequency interconnects and has been applied to the improvement in the efficiency of 3D field solvers, the modeling of lossy transmission lines, and design optimization [24,25,26,27]. Many popular macromodeling techniques exploit vector fitting (VF) [24,25] to build a rational function approximation for interconnect transfer functions. A novel, low-cost multivariate parametric macro modeling technique based on the kriging method for the residuals and poles of the reflection coefficients and the zeros of the transmission coefficients of microwave filters was proposed in [26]. A technique to derive macro-models of lumped-element networks for distributed microwave circuits by using system identification was presented in [27]. Nevertheless, both traditional 3D field solvers and those based on macromodeling optimization are becoming increasingly costly in terms of computer resources and simulation time, even more significantly so when the number of design choices and parameters increases [28].

In recent decades, powerful techniques of machine learning (ML) models such as artificial neural networks (ANNs) [29,30] and support vector machines (SVMs) [31,32] for the analysis and modeling of complex nonlinear problems have been developed as promising alternatives to the 3D field solver approach. ML models have high simulation speeds and do not need to fully understand the internal structure of modeling objects. Thus, S-parameter modeling technology based on ANNs has attracted increasing attention, and preliminary feasibility studies have been recently carried out [33,34,35,36]. While both SVMs and ANNs demonstrate efficacy in fitting, they have yet to be extensively applied in grid ground plane design parameters. Therefore, a method exhibiting low resource utilization and high convergence speed to achieve a balance between resource consumption and accuracy in predicting S-parameters would be beneficial to optimizing rigid-flex PCBs for InGaAs detection links.

In this study, we established a method for rapidly predicting the S-parameters of rigid-flexed PCBs based on a BP neural network for infrared InGaAs detection systems. We compared the generalized regression neural network (GRNN) model proposed in [36] and a traditional 3D field solver. The proposed method was implemented with a simple BP neural network structure, which can effectively control and prevent overfitting. Furthermore, the BP neural network only requires the storage of the weight matrix between each pair of layers, resulting in minimal memory usage. Compared with the traditional 3D field solver methods and the GRNN model, this approach significantly enhanced computational efficiency, reduced costs, and maintained the prediction error to less than 1%. This article is organized as follows: In Section 2, we briefly review the application and problems of rigid-flex PCBs in infrared InGaAs detection links and introduce BP networks. The details of our S-parameter modeling method for a rigid-flex board are also elucidated. In Section 3, we describe the data acquisition process and provide a detailed analysis of the prediction results. Finally, we provide the discussion and concluding thoughts in Section 4 and Section 5, respectively.

## 2. Prediction of Rigid-Flex Printed Circuit Board’s Scattering Parameters

### 2.1. Rigid-Flex Printed Circuit Boards and Problems of Infrared InGaAs Detection Links

**Figure 1:**
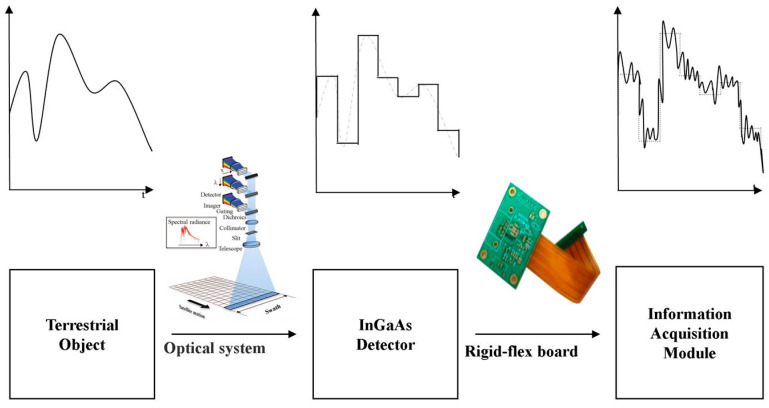
Infrared InGaAs detection link.

While collecting terrestrial object information from an optical system to an InGaAs detector, as a single pixel output is a fixed level value, the ideal readout circuit outputs an analog signal waveform of an amplitude-modulated square wave sequence. The amplitude of the latter reflects the frequency at which ground information can be obtained at that spatial resolution. Rigid-flex printed circuit boards combine rigid and flexible printed circuits. Due to the limitations on the position and size of infrared photodetectors in the structure, they cannot be directly integrated into the driver acquisition circuit board. Therefore, foldable rigid-flex printed circuit boards are used to complete the signal transmission between the infrared photodetectors and the information acquisition module, which results in a lightweight and compact device [37]. However, when the sensor’s output signal passes through the rigid-flex board, before reaching the information acquisition module, the ideal square wave sequence experiences distortion and produces unexpected noise, as shown in Figure 1.

**Figure 2:**
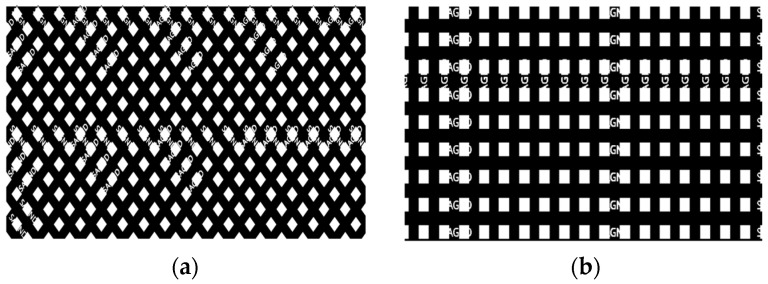
Two types of grid ground planes. (**a**) 45° uniform oblique grid. (**b**) Vertical crossed grid.

Circuit designers often opt for solid copper as the reference ground plane for rigid boards. Although solid copper provides superior shielding, using cross-hatched copper in flexible areas provides better bending and flexibility to the ground plane, ultimately reducing the probability of circuit damage and fracture. In addition, the cross-hatched copper results in better heat dissipation, solving the problem of uneven heating in solid copper, which causes PCB warpage. Consequently, cross-hatched copper is widely used in rigid-flex printed PCB manufacturing. In conventional cross-hatched reference planes, two types of grids are primarily used, i.e., 45° uniform oblique and vertical crossed grids, as illustrated in Figure 2. However, there is currently a lack of research on grid size, proportion, and form optimization, which often leads to poor shielding of the produced grid reference surface, which in turn can result in poor signal integrity and electromagnetic magnetic compatibility (EMC). The grid design affects the wiring’s parasitic capacitance and its projection area on the grid reference plane, where the latter also affects the former. In high-frequency circuits, it is essential to maintain the parasitic capacitance values as low as possible [38], as even small variations in this parameter can cause a significant frequency shift [39].

### 2.2. BP Neural Network

A BP neural network is an intelligent algorithmic model that simulates the thought process of the human brain. Learning inherent mappings from a large amount of data provides a powerful tool for solving complex nonlinear problems [40]. A BP neural network is a multi-layer feedforward network trained by an error backpropagation algorithm, and it is one of the most widely used and successful neural network models [41].

### 2.3. Modeling of Rigid-Flex PCB S-Parameters Based on BP Neural Network

Considering the mechanical structure and impedance matching problem of rigid-flex boards, the line width, thickness, length, and relative dielectric constant of the transmission line should be fixed. The main variation was in the design of the copper grid. At present, the design of the grid size and ratio of flexible boards is based on experience and lacks strict theoretical guidance, which may lead to more serious signal integrity and EMC problems. The above problem can further improve the signal transmission rate of rigid-flex circuit boards. To optimize the grid structure at the beginning of the design and improve the reliability of the device, this study analyzed the S-parameters based on a BP neural network to explore the effect of the grid copper design on signal integrity.

#### 2.3.1. Network Parameter Settings

**Figure 3:**
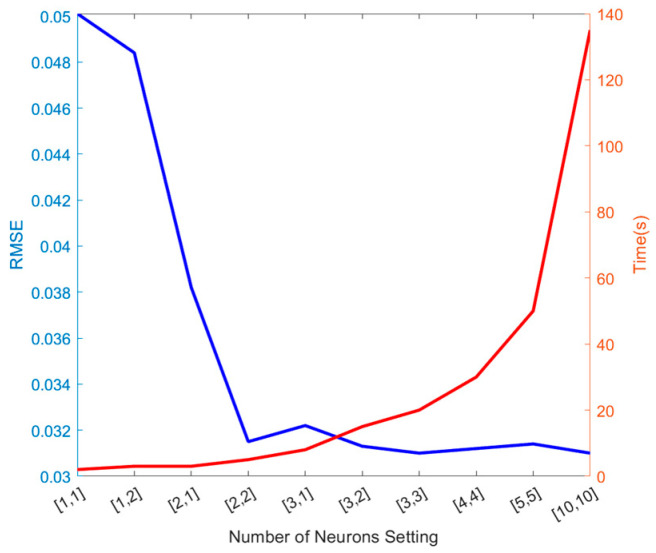
Number of neurons vs. RMSE and time.

The BP neural network architecture employed in this study comprised an input layer, an output layer, and two hidden layers. The dimension of the input layer was [1, 6], corresponding to the optimization variables for the grid ground plane. The dimension of the output layer was [1, 201], matching the number of frequency points. The S-parameters in the frequency domain were represented by a complex-valued function depending on frequency. To simplify the output, we decomposed it into real and imaginary parts, on which the network was trained separately to produce fitted curves at the output layer corresponding to the two parts at 201 frequency points. In Figure 3, the relationship between the settings for root mean square error (RMSE), time, and number of neurons in predicting the imaginary part of S_21_ is depicted. The horizontal coordinates are [number of neurons in the first hidden layer, number of neurons in the second hidden layer]. As shown in the figure, when the number of neurons increases, the RMSE does not decrease significantly, while the time cost increases. Therefore, we determined that utilizing two hidden layers with two neurons each provides an effective trade-off between model efficiency and accuracy. The network structure allows it to capture more complex nonlinear relationships and intricate nuances within the data, enhancing its ability to model the task while maintaining feasible resource requirements accurately.

**Figure 4:**
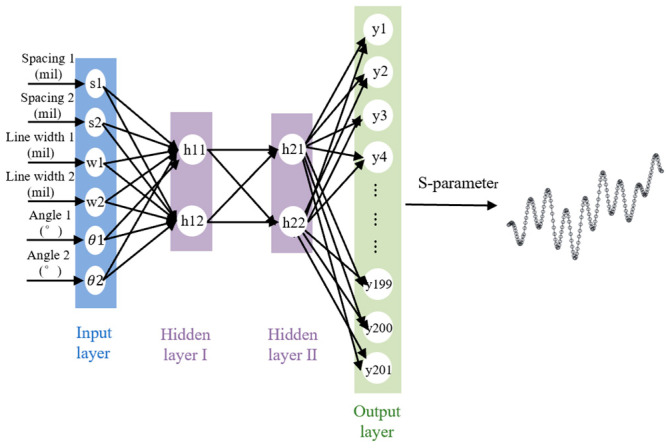
BP neural network model architecture.

Furthermore, the fitting effect of this configuration was deemed excellent. The loss function was set as the mean squared error (MSE), and the threshold was set to 0.001. The maximum number of training steps was 1000, and activation functions *f*1 and *f*2 were set as Tan-Sigmoid and Purelin functions, respectively. In Figure 4, the specific structure of the network prediction model is illustrated.

#### 2.3.2. Model Training

Several training algorithms are available for BP neural networks. Among the classical ones are the gradient descent algorithm and the Levenberg–Marquardt (LM) algorithm. The former is easy to implement, but its training convergence is slow, and it may even stop training prematurely. The LM algorithm combines the advantages of the gradient descent and Gauss–Newton methods, and its convergence speed is significantly higher than that of the gradient descent method while maintaining high accuracy. Therefore, the LM algorithm was adopted in this study to train the model. The error function is expressed as follows:

(1) $$E(w)=\frac{1}{2}\sum_{i=1}^{n}{\left(x_{i}-y_{i}\right)}^{2}=\frac{1}{2}\sum_{i=1}^{n}e_{i}^{2}(w)$$

where *n* represents the number of samples, ***w*** represents the weight vector, and $e_{i}^{2}(w)$ denotes the error value of the *i*th node in the output layer. The weight vector at the (*m* + 1)th iteration can be written as follows:

(2) $$w_{m+1}=w_{m}+\Delta w$$

where the weight increment is given as:

(3) $$\Delta w={\left[F^{T}(w)F(w)+\mu I\right]}^{-1}F^{T}(w)e(w)$$

The Jacobian matrix of the weights is calculated as follows:

(4) $$F(w)=\left[\begin{array}{cccc} \frac{\partial e_{1}(w)}{\partial w_{1}} & \frac{\partial e_{1}(w)}{\partial w_{2}} & \cdots & \frac{\partial e_{1}(w)}{\partial w_{n}}\\ \frac{\partial e_{2}(w)}{\partial w_{1}} & \frac{\partial e_{2}(w)}{\partial w_{2}} & \cdots & \frac{\partial e_{2}(w)}{\partial w_{n}}\\ \vdots & \vdots & & \vdots \\ \frac{\partial e_{N}(w)}{\partial w_{1}} & \frac{\partial e_{N}(w)}{\partial w_{2}} & \cdots & \frac{\partial e_{N}(w)}{\partial w_{n}} \end{array}\right]$$

where $w_{m}$ stands for the weight vector at the *m*th iteration, e(w) stands for the error vector, I stands for the identity matrix, and $\frac{\partial e_{N}(w)}{\partial w_{n}}$ stands for the partial derivative of the error vector over the weight vector.

## 3. Simulation

### 3.1. Data Acquisition and Analysis

**Figure 5:**
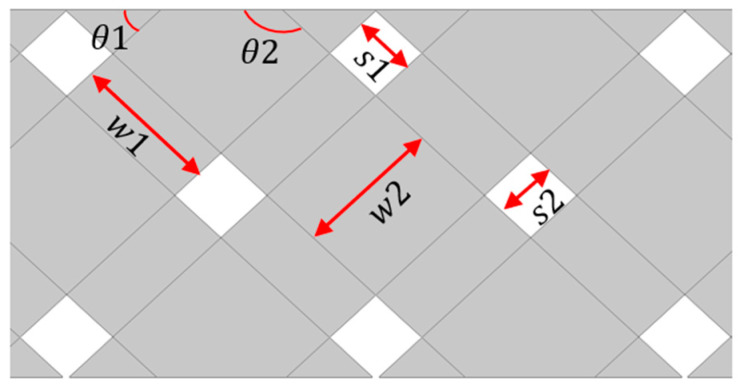
Line widths, spacings, angles of the reference ground plane copper.

An automated simulation platform was developed by using PowerSI (Cadence, San Jose, CA, USA) to obtain the S-parameter dataset. In this study, the ANN training was performed by using Matlab R2023b (MathWorks, Natick, MA, USA). The optimization parameters of the reference grid ground plane included line width, line spacing, and angle, as depicted in Figure 5. All simulations were performed on a personal computer equipped with an Intel Core i7-1360P (Intel Corporation, Santa Clara, CA, USA) CPU operating at 2.2 GHz with 32 GB memory.

When designing the reference ground grid copper as a 45° uniform oblique grid, θ1 and θ2 were set to 45° and 135°, respectively. Grid line width w1 was initially set to 6 mil and increased stepwise by 1 mil up to 15 mil, resulting in 10 different parameter settings grouped together. Moreover, w2, s1, and s2 were kept consistent within each group. However, when the reference ground grid copper was designed as a vertical crossed grid, the grid copper angles were altered to 90° and 180°. Spacings s1 and s2 and line widths w1 and w2 were set to be consistent with the 45° uniform oblique grid design. The specific parameter settings are listed in Table 1.

To verify the universality and robustness of the model proposed in this study for predicting the S-parameters of transmission lines in rigid-flex PCBs for aerospace payloads, a single-ended transmission line (A) and a differential transmission line pair (B) in the rigid-flex PCBs were selected respectively for simulation validation.

**Figure 6:**
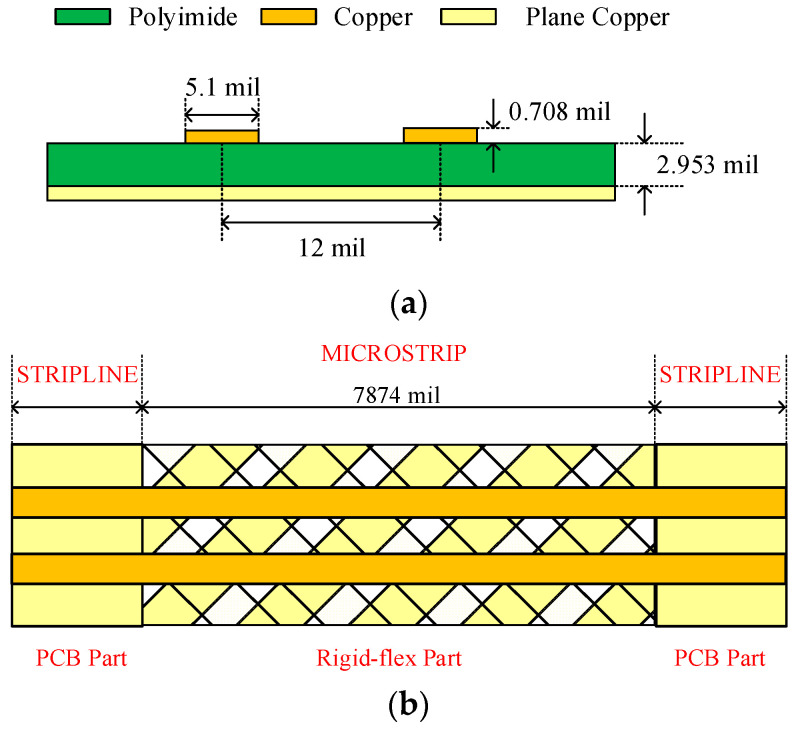
(**a**) Topology of Line A and Line pair B. (**b**) Topology of Line A and Line pair B on a rigid–flex PCBs.

The main parameter settings of Line A were as follows: the line width was 5.1 mil, the copper thickness was 0.708 mil, the transmission length was 7874 mil, the relative dielectric constant was 3.3, and the dielectric thickness was 2.953 mil. The differential transmission line pair (B) consisted of two Lines A with a 12 mil spacing, as shown in Figure 6.

The settings for all the simulations were consistent and included the following: the simulation frequency range was 1 kHz~2 GHz, and the linear scanning mode step size was 10 MHz; the frequency was divided into 201 points. 

**Figure 7:**
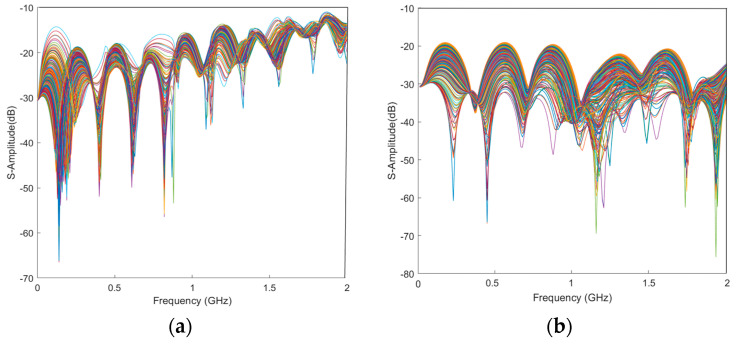
Reflection coefficients for 480 sets of different grid parameters. (**a**) The S_11_ of Single-ended transmission line A. (**b**) The S_DD11_ of differential transmission line pair B.

Finally, 480 different sets of S-parameter data were obtained with simulations for a single-ended transmission line (A) and a differential transmission line pair (B), respectively, as shown in Figure 7. To better verify the generalization ability of the proposed method and perform an unbiased evaluation, the samples obtained from the last 80 design parameters in Table 1 were selected as fixed test samples and were not used in model training. The first 400 samples in Table 1, each with an input dimension of 6 corresponding to the design parameters and an output dimension of 201 frequency points, were randomly divided into a test set and a validation set according to a 4:1 ratio, and a total of five experiments were conducted.

### 3.2. Prediction Results and Analysis

#### 3.2.1. Overfitting, Robustness and Generalization Ability Analysis

**Figure 8:**
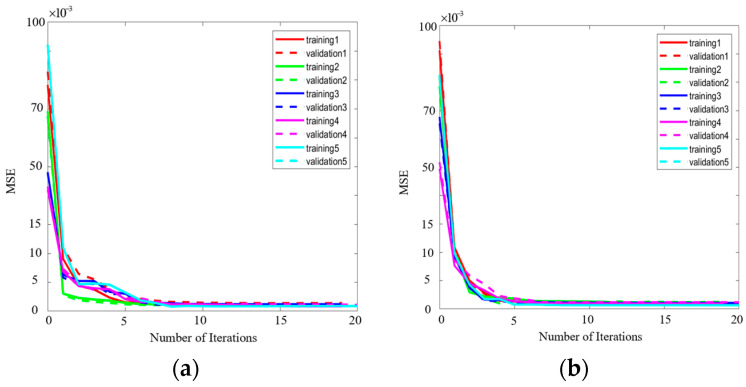
Performance of training and validation sets for 5 experiments. (**a**) Single-ended transmission line A. (**b**) Differential transmission line pair B.

Overfitting is a common issue in neural network training that leads to poor generalization performance on new data [42]. To investigate the susceptibility to overfitting of the proposed model, we reserved 20% of the samples in the training sets of the single-ended transmission line and differential transmission lines for validation data checking, respectively. To assess the generalization ability and robustness of the proposed model, five experiments were conducted with different training set compositions. As illustrated in Figure 8, despite variations in the training data, the mean MSEs on the training and validation sets converged to similar values and stabilized within each experiment. Notably, model performance improved, while the MSE curve on the training set did not further decrease, indicating that overfitting was effectively avoided. These results demonstrate that the proposed approach exhibits strong generalization capability and robustness, without being susceptible to overfitting issues. 

#### 3.2.2. Comparison of Goodness-of-Fit between BP Model and GRNN Model

**Figure 9:**
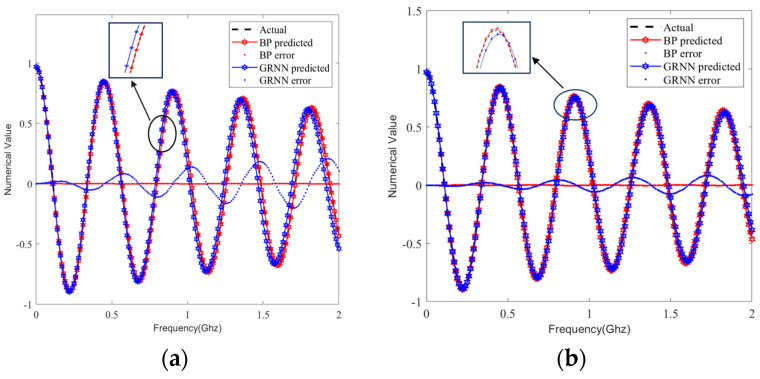
Comparison curves of real part predictions in 80 test sample sets. (**a**) Single-ended transmission line A’s S_21_. (**b**) Differential transmission line pair B’s S_DD21_.

**Figure 10:**
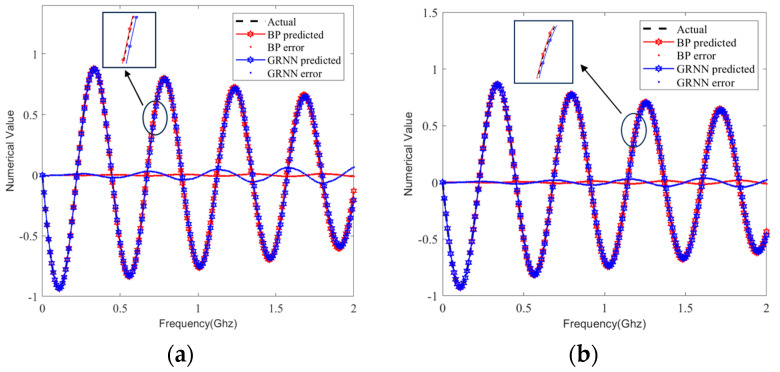
Comparison curves of imaginary part predictions in 80 test sample sets. (**a**) Single-ended transmission line A’s S_21_. (**b**) Differential transmission line pair B’s S_DD21_.

**Figure 11:**
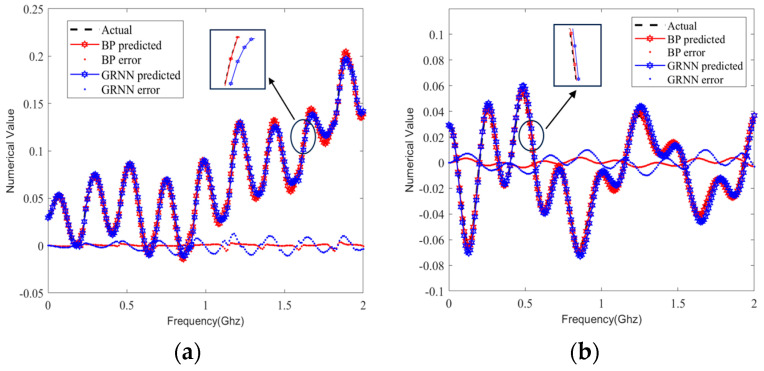
Comparison curves of real part predictions in 80 test sample sets. (**a**) Single-ended transmission line A’s S_11_. (**b**) Differential transmission line pair B’s S_DD11_.

**Figure 12:**
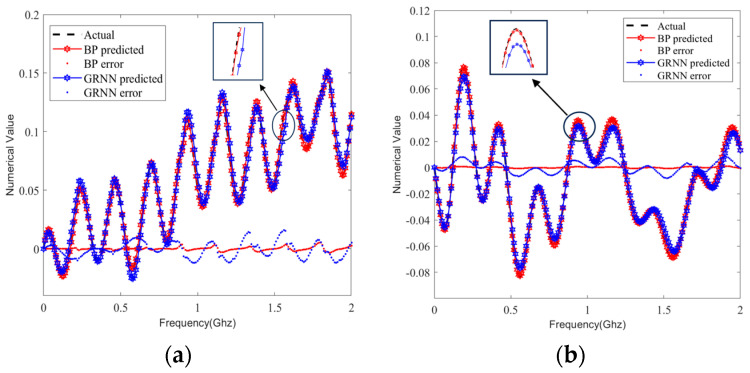
Comparison curves of imaginary part predictions in 80 test sample sets. (**a**) Single-ended transmission line A’s S_11_. (**b**) Differential transmission line pair B’s S_DD11_.

We selected the same sample set from the test set and predicted the S-parameters by using the BP and GRNN models; the prediction curves are compared in Figure 9, Figure 10, Figure 11 and Figure 12. It can be clearly seen that the S-parameters predicted by using the BP model were closer to the actual values than those predicted by the GRNN model in both the real and imaginary parts.

**Figure 13:**
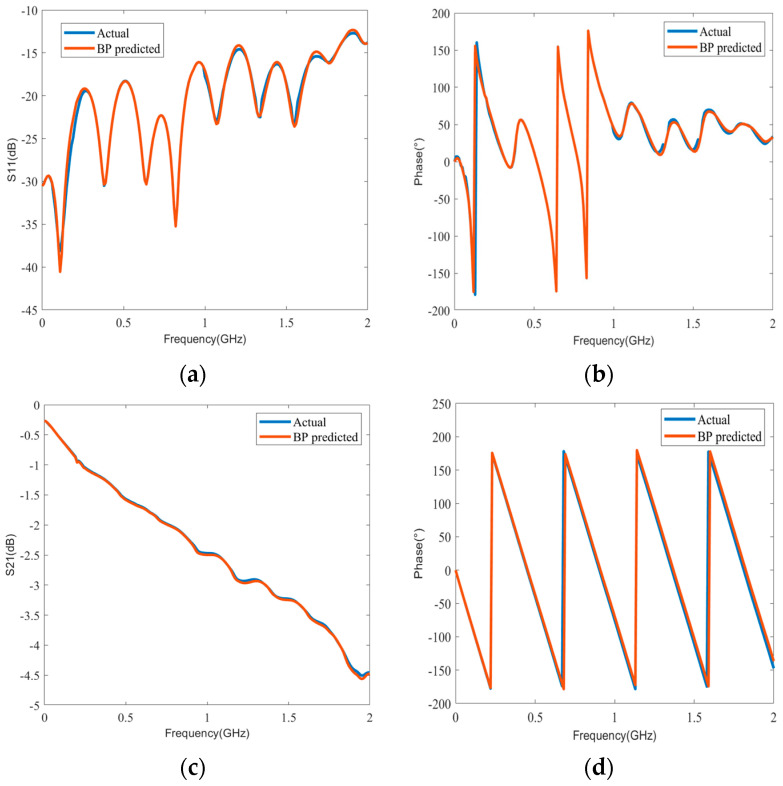
(**a**) Comparison results of amplitude prediction for *S*_11_. (**b**) Comparison results of phase prediction for S_11_. (**c**) Comparison results of amplitude prediction for *S*_21_. (**d**) Comparison results of phase prediction for S_21_.

The amplitude and phase can also be calculated from the real and imaginary parts, which can help us understand the physical meaning of the S-parameters more intuitively. According to the above comparison results, we used the proposed BP model for curve fitting. A test sample was randomly selected, and Figure 13a–d illustrates the differences between the actual and predicted values of the amplitude and phase for S_11_ and S_21_, respectively. It can be seen from the four sub-figures that the predicted values of the amplitude and phase fit well with the actual values, which demonstrates the superior prediction performance of the proposed method. 

Comparing curves from a single test sample is not sufficient to demonstrate the accuracy and robustness of a method. For nonlinear regression models, the goodness-of-fit is a critical evaluative metric that quantifies how closely the model aligns with the observed data. Greater goodness-of-fit values indicate that the regression function more accurately describes the data. 

A common approach to assessing the goodness-of-fit is calculating the adjusted R^2^ [43], which accounts for model complexity and degrees of freedom to mitigate overfitting. This metric ranges from 0 to 1, where values nearing 1 indicate that the model adequately explains variation in the data, while values approaching 0 denote poor explanatory power. Moreover, the goodness-of-fit analysis was expanded to include the RMSE, providing additional insights into model performance from a different perspective. While the adjusted R^2^ quantifies the variance explained by the model, the RMSE measures residual differences between predicted and actual values. Evaluating nonlinear regression models by using the adjusted R^2^ and RMSE provides a complementary quantification of model fit quality.

**Figure 14:**
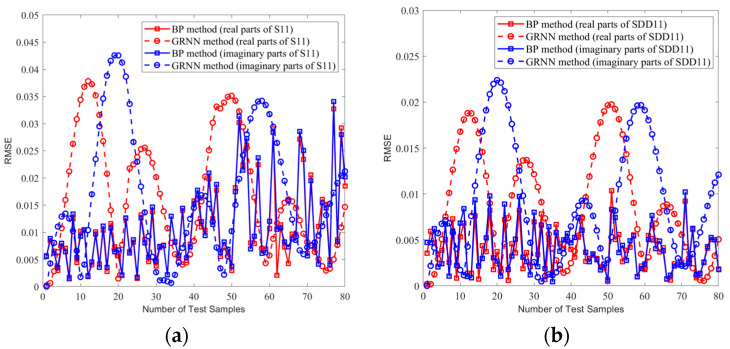
The RMSE of the real and imaginary parts of the same 80 test sample sets. (**a**) Single-ended transmission line A. (**b**) Differential transmission line pair B.

**Figure 15:**
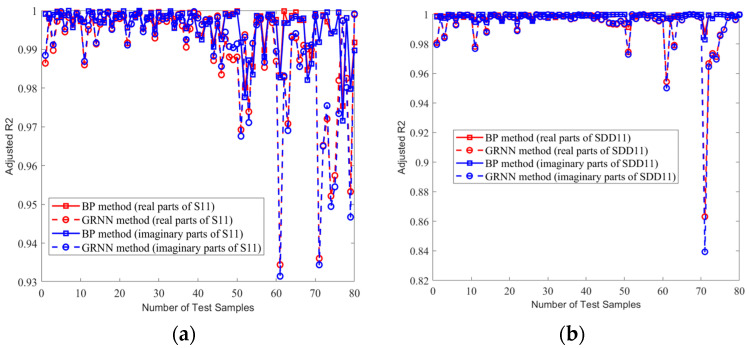
The adjusted R^2^ of the real and imaginary parts of the same 80 test sample sets. (**a**) Single-ended transmission line A. (**b**) Differential transmission line pair B.

Therefore, we compared the prediction performance of the proposed BP model and the GRNN model by evaluating the goodness-of-fit metrics in all 80 test samples. In Figure 14 and Figure 15, we show the comparison of averaging the RMSE and the adjusted R^2^, respectively, of the real and imaginary parts of the 80 identical test sets in one experiment for the single-ended transmission line and the differential line pair. It can be seen that our method outperformed the GRNN model in 80 samples, as evidenced by smaller RMSEs and adjusted R^2^ values closer to 1. 

To further demonstrate the robustness and high fit of the proposed method, five experiments were conducted as described in Section 3.1. The quantitative analysis summarized in Table 2 and Table 3 reinforced the superior goodness-of-fit of the BP model, as the average adjusted R^2^ was closer to 1 in the five experiments on 80 test sets. 

#### 3.2.3. Time Efficiency

The time periods required to obtain 40 and 80 S-parameter curves by using PowerSI software simulation were 200 min and 400 min, respectively. However, by training the two-port network model (the real and imaginary parts of S_11_, S_21_, S_12,_ and S_22_) once with the BP model and predicting the 40 and 80 S-parameter curves, these were reduced to 51.3 s and 51.7 s, respectively. The GRNN model took 68.2 s and 68.7 s, respectively, to do so. It is evident that obtaining S-parameters by using the neural network-trained model significantly reduces the time cost of evaluating the S-parameters, as the prediction time was almost negligible even as the number of S-parameter curves increased, while the time necessary when using traditional simulation software increased linearly. Furthermore, the training and prediction efficiency of the BP model was higher than that of the GRNN model. Therefore, our method can improve the efficiency of iteratively optimized rigid-flex PCBs.

#### 3.2.4. Errors in Eye Height and Eye Width

**Figure 16:**
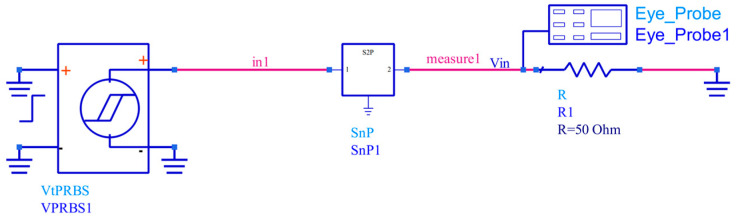
Transient simulation is built in the ADS.

To further validate the modeling accuracy, time–domain transient simulations were performed for a single-ended transmission line by using the S-parameters predicted from the proposed BP model, the GRNN model [36], and the corresponding test samples, respectively. A pseudorandom binary sequence (PRBS) with a code length of 255 was applied as the excitation source at 1 Gbps with 0.25 ns rise/fall time. The transient simulations were implemented in ADS, as shown in Figure 16, to obtain the simulated eye diagrams. A total of 80 test samples of predicted and actual S-parameters were used in the simulations. Eye height and eye width errors were extracted from the simulated eye diagrams and analyzed. Employing time–domain transient simulations allowed us to quantitatively analyze the modeling accuracy with industry-standard statistical eye diagram parameters.

**Figure 17:**
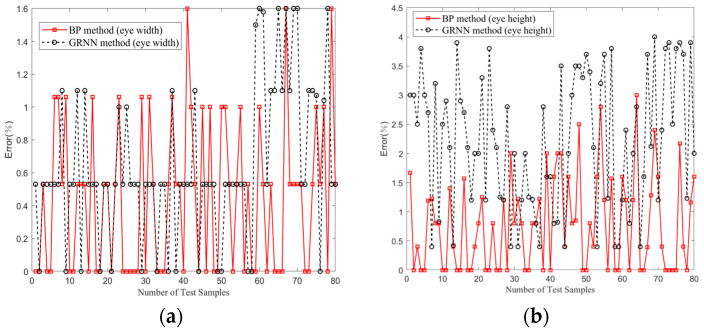
Comparison of error trends by the BP and GRNN methods in 80 test sample sets. (**a**) Eye width. (**b**) Eye height.

The results demonstrate excellent concordance between the eye diagrams predicted by using the proposed model and those obtained with simulations. Notably, the errors associated with the eye width and eye height parameters were 0.44% and 0.78%, respectively. These error values represent a significant reduction, being merely 0.68 times and 0.35 times the corresponding errors obtained with the GRNN model. In Figure 17, the trends of eye height and eye width errors for 80 sets of test samples in one experiment are reported. In Table 4, the simulated mean, predicted mean, and error mean of eye height and eye width in the experiment are listed.

## 4. Discussion

Considering the significance of rigid-flex PCBs in InGaAs detection links for weak infrared detection signals, we investigated the use of BP neural networks to rapidly predict the S-parameters of the grid ground plane of rigid-flex PCBs. We evaluated the results by using robustness, goodness-of-fit, generalization ability, time efficiency, and eye width and eye height errors as performance metrics. The results indicate that the proposed BP neural network can accurately reconstruct the nonlinear behavior of the grid ground plane design for rigid-flex PCBs over the entire frequency range. The constructed neural network model can effectively replace simulations in obtaining S-parameters with good prediction performance.

As presented in Section 3, in-depth research was conducted in order to demonstrate the choice of the model and its accuracy and time efficiency compared with other prediction models. The GRNN method has been used to predict the HBT scattering parameters at different temperatures [36]. However, unlike the approach in [36], our method can predict values within a certain frequency range instead of a single frequency value. From the perspective of the goodness-of-fit, the RMSE of the BP model was smaller (see Table 2), and the adjusted R^2^ was maximally 0.06 larger than that of the GRNN model (see Table 3). It is evident from the results that using an ANN to predict the S-parameters of the differential transmission line pair was more effective than using it to predict those of the single-ended transmission line. Based on the results of the eye width and eye height errors, the BP model improved them by 0.21% and 1.44%, respectively (see Table 4). In terms of time efficiency, the BP model also showed faster performance. 

In addition, we demonstrated the potential of our neural network for designing the grid ground plane by comparing it with a traditional 3D field solver. This confirms the effectiveness of neural networks in electromagnetic modeling and parameter prediction, as previously shown in other studies [29,30,31,32,33,34,35,36]. Given its predictive capability, the model represents a powerful tool for system-level simulation and optimization, eliminating the need for tedious and repetitive simulation processes and saving significant time and computational resources. This result meets the current industrial demand for efficient design optimization methods and provides new ideas for future research in the field of rigid-flex PCBs and InGaAs detector applications. However, despite the results obtained in this study, there is still room for improvement. In future work, we will focus on using the higher-efficiency model to solve more complex problems, thereby broadening its applicability.

## 5. Conclusions

In the aerospace field, large-array InGaAs focal plane detectors for quantitative payload applications have experienced rapid development. Consequently, there is an increased demand for precise noise control and quantization of high-speed weak signals. Detailed analysis of each detection system component is necessary to achieve this goal. In this study, we designed a BP neural network to predict the scattering parameters of rigid-flex PCBs for InGaAs infrared detection links. 

The simulation verification and analysis results demonstrate that the BP network model can achieve high-precision scattering parameter prediction. Moreover, the efficiency of this model was found to be 463 times higher than that of a traditional 3D field solver when 80 S-parameters were obtained. It also exhibited 25% faster performance and 30% and 65% smaller eye errors than the GRNN model. 

This method effectively optimizes the grid copper design in early rigid-flex PCB layout stages, substantially reducing the design cycle time. It also improves design quality for aerospace quantitative payloads. The findings of this study represent a foundation for future infrared detection systems requiring high sensitivity and low noise. They can also provide technical support to expand the application prospects of infrared weak-spectrum detection. It is recommended that other influencing factors be considered to improve prediction accuracy in future research. Moreover, extending this modeling approach to other vital electrical parameters would enable more comprehensive PCB evaluation and optimization.

## Figures and Tables

**Table 1:** Settings of mesh parameters.

Group Number	w1 (mil)	w2 (mil)	s1 (mil)	s2 (mil)	θ1 (°)	θ2 (°)
1	6~15	6	6	15	45	135
2	6~15	7	7	14	45	135
⋮	⋮	⋮	⋮	⋮	⋮	⋮
10	6~15	15	15	6	45	135
11	6~15	6	15	15	45	135
12	6~15	7	14	14	45	135
⋮	⋮	⋮	⋮	⋮	⋮	⋮
20	6~15	15	6	6	45	135
21	6~15	6	6	15	90	180
22	6~15	7	7	14	90	180
⋮	⋮	⋮	⋮	⋮	⋮	⋮
30	6~15	15	15	6	90	180
31	6~15	6	15	15	90	180
32	6~15	7	14	14	90	180
⋮	⋮	⋮	⋮	⋮	⋮	⋮
40	6~15	15	6	6	90	180
41	6~15	6	8	14	45	135
42	6~15	8	10	12	45	135
43	6~15	10	12	10	45	135
44	6~15	12	14	8	45	135
45	6~15	6	8	14	90	180
46	6~15	8	10	12	90	180
47	6~15	10	12	10	90	180
48	6~15	12	14	8	90	180

**Table 2:** Average RMSE over five experiments.

	BP	GRNN
Real parts of S_11_	0.0107	0.0168
Imaginary parts of S_11_	0.0109	0.0165
Real parts of S_21_	0.0210	0.0214
Imaginary parts of S_21_	0.0219	0.0205
Real parts of S_DD11_	0.0042	0.0086
Imaginary parts of S_DD11_	0.0041	0.0090
Real parts of S_DD21_	0.0106	0.0130
Imaginary parts of S_DD21_	0.0105	0.0138

**Table 3:** Average adjusted R^2^ over five experiments.

	BP	GRNN
Real parts of S_11_	0.9961	0.9899
Imaginary parts of S_11_	0.9958	0.9899
Real parts of S_21_	0.9968	0.9928
Imaginary parts of S_21_	0.9974	0.9932
Real parts of S_DD11_	0.9987	0.9932
Imaginary parts of S_DD11_	0.9987	0.9926
Real parts of S_DD21_	0.9996	0.9971
Imaginary parts of S_DD21_	0.9997	0.9972

**Table 4:** Comparison of eye width and eye height obtained by different methods.

	Error of Eye Width (%)	Error of Eye Height (%)
BP method	0.44	0.78
GRNN method	0.65	2.22

## Data Availability

Data generated during the study are contained within the article.

## Abbreviations

The following abbreviations are used in this manuscript:

3D	Three-dimensional
ANN	Artificial Neural Network
BP	Backpropagation
EDA	Electronic Design Automation
EMC	Electromagnetic Magnetic Compatibility
EMI	Electromagnetic Interference
GRNN	Generalized Regression Neural Network
LM	Levenberg–Marquardt
ML	Machine Learning
MSE	Mean Squared Error
RMSE	Root Mean Squared Error
PCB	Rigid-flex Printed Circuit Board
PRBS	Pseudo-random Binary Sequence
S-parameters	Scattering Parameters
SVM	Support Vector Machine
VF	Vector Fitting
